# Hot Deformation Behavior and Microstructural Evolution Based on the Processing Map of Dual-Phase Mg-Li Based Alloy

**DOI:** 10.3390/ma15031022

**Published:** 2022-01-28

**Authors:** Jiangtao Guo, Shengli Guo, Yazhao Shen, Defu Li

**Affiliations:** 1 State Key Laboratory of Nonferrous Metals and Processes, GRINM Group Co., Ltd., Beijing 101407, China; gjttju@163.com (J.G.); shenyazhao_1994@163.com (Y.S.); lidf@grinm.com (D.L.); 2 GRIMAT Engineering Institute Co., Ltd., Beijing 101407, China; 3 General Research Institute for Nonferrous Metals, Beijing 100088, China; 4 GRINM Group Corporation Limited, Beijing 100088, China

**Keywords:** Mg-Li-Al-Zn-Si alloy, hot compression, constitutive model, processing map, dynamic recrystallization

## Abstract

The deformation behavior of the as-extruded Mg-Li-Al-Zn-Si alloy was studied by conducting a hot compression test, with a temperature range of 180–330 °C and a strain rate range of 0.01–10 s^−1^. The constitutive relationship of flow stress, temperature, and strain rate was expressed by the Zener–Hollomon parameter and included the Arrhenius term. By considering the effect of strain on the material constants, the flow stress at 240–330 °C could be precisely predicted with the constitutive equation (incorporating the influence of strain). A processing map was established at the strain of 0.7. The unsafe domains that are characterized by uneven microstructures were detected at low temperatures (<230 °C) or high temperatures (>280 °C), with high strain rates (>1 s^−1^). The optimum hot deformation region was obtained at a medium temperature (270–300 °C), with a peak power dissipation efficiency of 0.44. The microstructural evolution in different domains is investigated. The unstable domains are characterized by a non-uniform flow behavior and uneven microstructure. The observation showed that the dynamic recrystallization (DRX) process could easily occur at the safe domain with high power dissipation efficiency. For the α-phase, some features of continuous dynamic recrystallization can be found. The triple points serve as prominent nucleation sites for the β-phase DRX grains and the growth in the grains was carried out by subgrain boundary migration. The microstructure exhibits characteristics of discontinuous dynamic recrystallization.

## 1. Introduction

Mg-Li alloy, usually referred to as ultra-light Mg alloy, is currently considered to be the lightest metal structural material. The density of Mg-Li alloy is 1/4–1/3 lower than that of the Mg alloy, and is only about 1.30–1.65 g/cm^3^ [1]. Additionally, owing to properties such as superior specific strength, high specific stiffness, and high damping capacity, the Mg-Li alloy is widely used in aerospace, the military, and the 3C industry [2]. Traditional Mg alloys have poor formability due to their hexagonal, close-packed (HCP) structure, which leads to poor ductility and limits their applications. Below 22 °C, the plastic deformation of polycrystalline magnesium relies on basal slip and twinning. With the addition of the Li element, not only the density of the alloy but also the c/a axial ratio decreases. The decrease in c/a could cause non-basal slip (pyramidal <c + a> slip) to occur at the initial stage of deformation [3]. Based on the Mg-Li phase diagram, the crystal structure of the alloy that consists of less than 5.5% Li content has an HCP structure, like traditional Mg alloys. The alloy containing 5.5–11.5% Li content is composed of an HCP a-Mg phase and a BCC β-Li phase, which have a great advantage with regard to ductility. Once the content of Li is over 11.5 wt.%, there will be a single BCC β-Li phase [4]. Among various Mg-Li alloys, the dual-phase alloys keep a superior balance of strength and ductility [5]. However, the relatively low strength and poor thermal stability become obstacles when it comes to their application.

Plenty of studies have shown that alloying and plastic deformation strengthening can significantly improve the mechanical properties and corrosion resistance of Mg-Li alloys. Research has shown that Al reinforces the mechanical strength of the Mg-Li matrix [6]. Li et al. studied the solid solution treatment of Mg-10.1Li-4.8Al. The results indicated that the tensile strength of a sample was increased from 127 MPa (as-cast sample) to 250 MPa (T4 sample) by adjusting the solution parameters, and the corrosion resistance of the T4 sample was also improved simultaneously [7]. With the addition of Zn, the corrosion resistance of the alloy is further enhanced. Anna et al. have investigated the influence of bimodal grain size distribution on the corrosion resistance of Mg-4Li-3Al-1Zn. The study of extruded and annealed alloy showed the major corrosion mechanism that occurred between high-angle grain boundaries and grain interiors [8]. A further addition of elemental Si can produce particle reinforcement, which can improve the thermal stability of the Mg-Li alloy. Shao et al. have researched the microstructural evolution and mechanical properties of Mg-10Li-3Al-3Zn-0.25Si during the thermal cycling process. After the thermal cycling process, the strength of the alloy was decreased by 6–8%, while the elongation was enhanced by about 65% [9].

There are quite a few studies on the hot deformation behavior of the Mg-Li alloys [10,11]. Due to their low stacking fault energy, dynamic recrystallization (DRX) is the main softening mechanism of Mg alloys during the hot deformation process [12]. Yang et al. have discussed the microstructural evolution of the LA93-xSr alloy during extrusion. To account for the differences in structure between α-Mg and β-Li, the dominant mechanism of the DRX in the α-Mg phase is continuous dynamic recrystallization (CDRX) process. The existence of α-Mg and Al_4_Sr phases promote the discontinuous dynamic recrystallization (DDRX) of the β-Li phase [13]. The DRX process achieves refinement of the grains and the well-distribution of phases in the microstructure. Therefore, studying the influence of the DRX process on deformation parameters is beneficial to the understanding of the microstructural evolution of duplex phase Mg-Li alloys.

In this study, hot compression tests were conducted at various temperatures and strain rates. The hot deformation behavior and microstructural evolution were studied. One purpose of this study is to construct the constitutive relation and the processing map of the as-extruded Mg-Li-Al-Zn-Si alloy to optimize conditions for hot working, and the other is to improve the understanding of nucleation mechanisms of the DRX process in two phases.

## 2. Materials and Methods

The material used in the experiment was an as-extruded rod from an industrial company (Sifang ULW Co., Ltd., Xi’an, China), and the chemical composition of Mg-Li-Al-Zn-Si alloy is given in Table 1.

The cylindrical specimens for hot compression tests, which were 10 mm in diameter and 15 mm in height, were machined from the extruded rod along the extrusion direction. Isothermal hot compression tests were performed on a thermo-mechanical simulator (Gleeble-3500) (DATA SCIENCES INTERNATIONAL, INC, St. Paul, MN, USA). The compression tests were performed at temperatures that ranged from 180 to 330 °C, with strain rates that ranged from 0.01 to 10 s^−1^. Both ends of the specimen were padded with graphite sheets to reduce the effect of friction. The specimens were heated to the predetermined temperature at a heating rate of 10 K/s and held for 1 min to eliminate thermal gradients. Subsequently, the specimens were compressed to a true strain of about 0.7 and immediately followed by water quenching to maintain the deformed microstructure.

For microstructural observation, the deformed specimens were cut along the direction of compression. The surfaces were mechanically polished and boiled in the solution of 1 mL HNO_3_ + 1 mL CH_3_COOH + 3 g C_2_H_2_O_4_ + 100 mL H_2_O for the observation by an optical metallographic technique and a scanning electron microscope (SEM, JSM-7001F-JOEL, Tokyo, Japan) equipped with an Oxford energy-dispersive X-ray spectroscope (EDS, Aztec, Oxford Instruments, Oxford, UK). To confirm the phase composition of the alloy, X-ray diffraction (XRD) was performed using a Bruker D8 Advance diffractometer (Bruker, Karlsruhe, Germany) operating at 40 kV and 40 mA with Cu Ka radiation. Transmission Electron Microscope (TEM) characterization was carried out with a Tecnai-G2-F20-FEI transmission electron microscope (FEI, Hillsboro, OR, USA) operated at 200 kV. The samples with a 3 mm diameter for TEM investigation were prepared by mechanical grinding to a thickness of 50 μm, and then thinned using twin-jet electropolishing with a solution of HClO_4_ (10%) + C_2_H_5_OH (90%) at the temperature of −30 °C. The voltage and current used were 80 V and 90 mA, respectively.

## 3. Results and Discussion

### 3.1. Microstructure of As-Extruded Mg-Li-Al-Zn-Si Alloy

**Figure 1:**
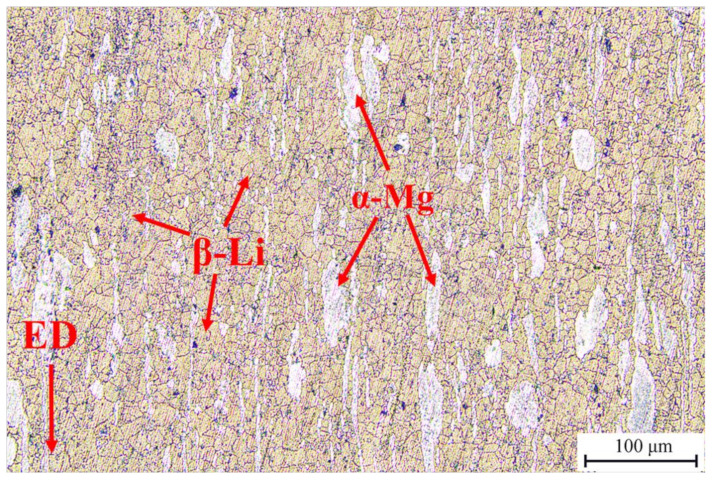
Microstructure of as-extruded Mg-Li-Al-Zn-Si alloy in longitudinal section.

The optical micrograph of the as-extruded Mg-Li-Al-Zn-Si alloy is shown in Figure 1. The typical duplex phase consists of α-Mg with an HCP structure and β-Li with a BCC structure. The dark areas are the β-Li matrix phases with equiaxed grains, and the bright areas are the α-Mg phases, which are lath-shaped and run parallel to the extrusion direction (ED, as the arrow shown in Figure 1). The average grain size of the β-phase is about 10.71 μm.

**Figure 2:**
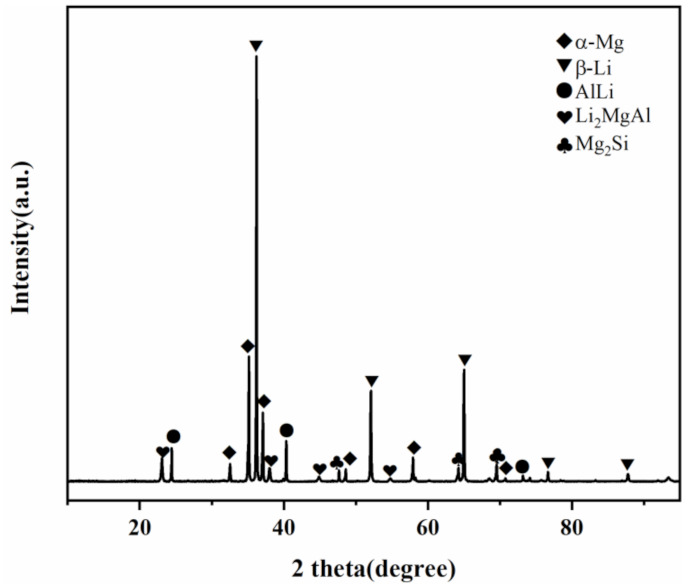
XRD patterns obtained for as-extruded Mg-Li-Al-Zn-Si alloy.

Figure 2 shows the XRD pattern measurements of the as-extruded Mg-Li-Al-Zn-Si alloy. Due to the addition of alloying elements, there are several extra phases. The α-Mg and β-Li are the major phases of the alloy. With the addition of Al, AlLi and Li_2_MgAl despites in the matrix [14,15]. After the introduction of Si, the Mg_2_Si phase remains and is stable during the thermal cycling process. Furthermore, its existence is beneficial to the thermal stability of the alloy [9].

**Figure 3:**
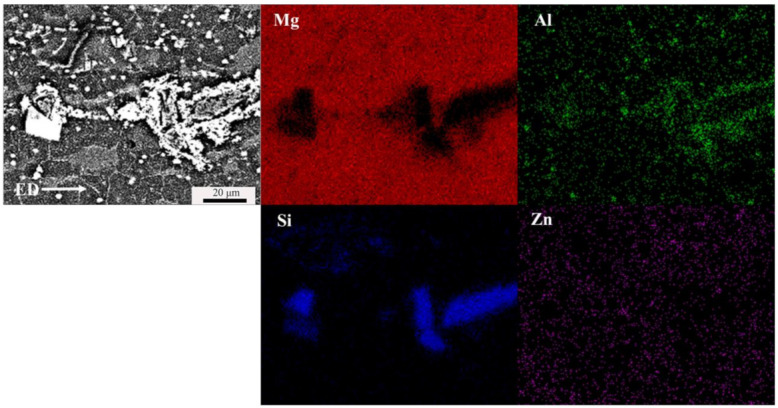
SEM image and EDS chemical mapping of as-extruded Mg-Li-Al-Zn-Si alloy.

Figure 3 shows the element distribution maps. With the high solid solubility in Mg-Li alloy, Al and Zn elements mostly exist in the matrix, inducing the increase in lattice distortion energy, which results in the obvious inhibitory effect of dislocation movement to improve the strength of materials [16]. The AlLi and Li_2_MgAl phases are dispersed in the matrix as granular bright phases. The Mg_2_Si phases are spread in grain boundaries and phase boundaries.

### 3.2. True Stress–Strain Curves

**Figure 4:**
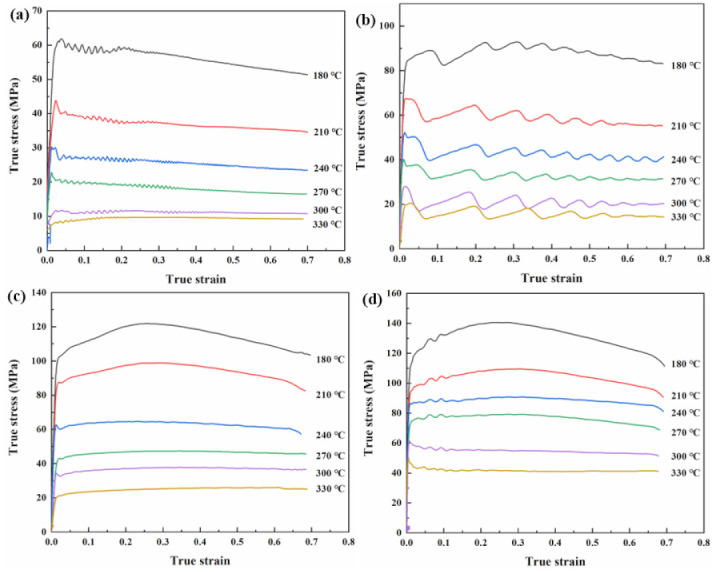
True stress–strain curves of Mg-Li-Al-Zn-Si alloy deformed to a strain of 0.7 at strain rates of (**a**) 0.01 s^−1^, (**b**) 0.1 s^−1^, (**c**) 1 s^−1^ and (**d**) 10 s^−1^ with different temperatures.

Figure 4 shows the smoothed true stress–strain curves of the Mg-Li-Al-Zn-Si alloy, deformed under temperatures ranging from 180 to 330 °C withstrain of 0.7, and at strain rates from 0.01 s^−1^ to 10 s^−1^. It is revealed that the flow stress is obviously affected by the deformation temperature during hot compression deformation [17,18]. With an increased deformation temperature or decreased strain rate, the flow stress decreased. Notably, the flow stress was increased rapidly at a very early stage of the deformation, and can be related to the work hardening caused by dislocation multiplication and tangle. With an increased strain, the flow stress slowly reaches a peak value. The internal distortion energy of the metal accumulates to a certain extent with an increased dislocation density. Furthermore, the softening mechanisms, such as dynamic recovery (DRV) and dynamic recrystallization (DRX), play an important role. It is worth noting that a dynamic equilibrium mode between work hardening and dynamic softening is established at the strain rate of 0.01 and 0.1 s^−1^. The multiple peaks caused by the DRX cycle occurred before a steady-state arrived. The DRX cycle phenomenon has been described by a multi-phase-field model [19,20] and has been observed in other research projects [21,22].

### 3.3. Constitutive Equation

There is an interactive relationship among the flow stress (*σ*), deformation temperature (*T*), and strain rate ($\dot{\varepsilon }$) during the hot deformation process. The study of Sellars suggests that *σ* is determined by *T* and $\dot{\varepsilon }$, which can be expressed as [23]:

(1) $$Z=\dot{\varepsilon }\exp\left(\frac{Q}{RT}\right)=A{\left[\sinh\left(\alpha \sigma \right)\right]}^{n}$$

Equation (1) is also known as the Arrhenius equation, where *Z* is the Zener–Holloman parameter, and its physical meaning is the deformation rate factor of temperature compensation: $\dot{\varepsilon }$ is the strain rate (s^−1^), *Q* is the deformation activation energy (kJ·mol^−1^), *R* is the universal gas constant (*R* = 8.314 J·mol^−1^·K^−1^), T is the deformation temperature (K), *σ* is the true stress (MPa), *A*, *α*, and *n* are the temperature-independent material constants. Equation (2) is based on the Taylor expansion [24]:

(2) $$Z=\dot{\varepsilon }\exp\left(\frac{Q}{RT}\right)=\left\{\begin{array}{l} A_{1}\sigma^{n_{1}},\alpha \sigma <0.8\\ A_{2}\exp\left(\beta \sigma \right),\alpha \sigma >1.2 \end{array}\right.$$

where *α* = *β*/*n*_1_. Taking the natural logarithm of Equations (1) and (2), respectively, Equations (3) and (4) were then obtained:

(3) $$\ln Z=\ln\dot{\varepsilon }+\frac{Q}{RT}=\ln A+n\ln\left[\sinh\left(\alpha \sigma \right)\right]$$

(4) $$\ln Z=\ln\dot{\varepsilon }+\frac{Q}{RT}=\left\{\begin{array}{l} \ln A_{1}+n_{1}\ln\sigma ,\alpha \sigma <0.8\\ \ln A_{2}+\beta \sigma ,\alpha \sigma >1.2 \end{array}\right.$$

Take the derivative of 1/*T* on both sides of Equation (3):

(5) $$\frac{Q}{R}=n\cdot {\left.\frac{\partial \ln\left[\sinh\left(\alpha \sigma \right)\right]}{\partial \left(\frac{1}{T}\right)}\right|}_{\dot{\varepsilon }},$$

**Figure 5:**
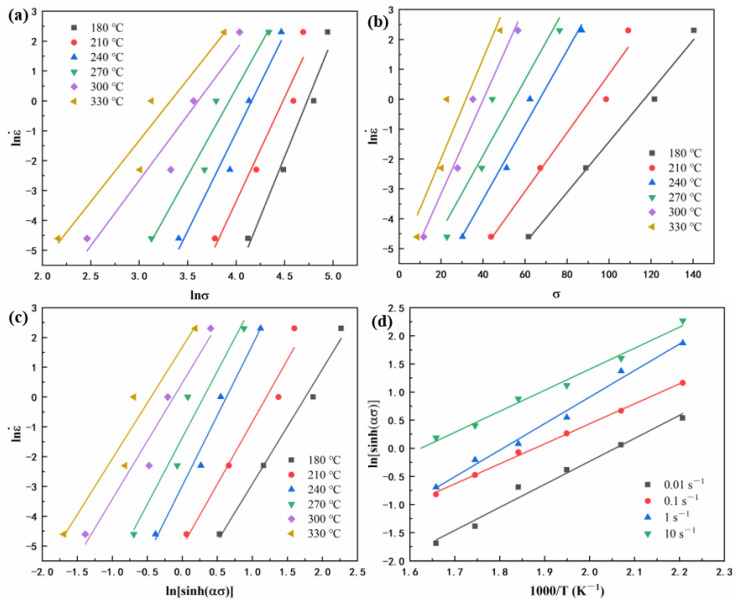
Relationship between: (**a**) ln$\dot{\varepsilon }$ and lnσ, (**b**) ln$\dot{\varepsilon }$ and σ, (**c**) ln[sinh(*ασ*)] and ln$\dot{\varepsilon }$, (**d**) 1000/T and ln[sinh(*ασ*)].

The discrete flow stress data at strains ranging from 0.01 to 0.69, with an interval of 0.0004, are used to calculate the parameters mentioned above. The lnσ-ln$\dot{\varepsilon }$, σ-ln$\dot{\varepsilon }$, ln[sinh(*ασ*)]-ln$\dot{\varepsilon }$, and 1000/T-ln[sinh(*ασ*)] curves shown in Figure 5 were processed and linearly fitted according to the experimental data based on Equations (3)–(5).

The slopes of the corresponding lines shown in Figure 4 represent n_1_, β, n, *Q*/Rn, respectively. The mean values of the slopes are taken to evaluate the values of n_1_, β, n, *Q*/Rn, which are 5.981, 0.126, 4.143, 4.0251. The values of α and *Q* can be calculated as 0.0211 and 138.643 kJ·mol^−1^, respectively.

Substitute values of n, α, *Q* into Equation (3):

(6) $$\ln Z=\ln A+4.143\ln\left[\sinh\left(0.0211\sigma \right)\right],$$

**Figure 6:**
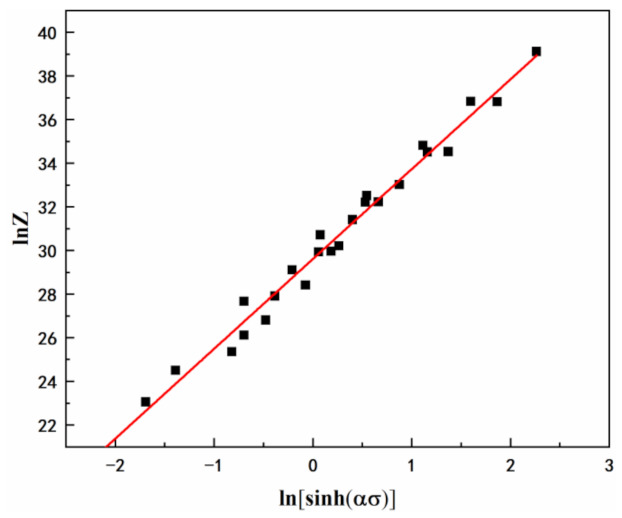
Relationship between ln*Z* and ln[sinh(*ασ*)].

Figure 6 shows a linear relationship between ln*Z*-ln[sinh*ασ*] based on Equation (6). ln*A* is the intercept of Figure 6, which can be obtained as ln*A* = 29.6. Therefore, the value of *A* could be obtained as *A* = e^29.6^.

According to the above values, the hyperbolic sine equations of the peak stress for the as-extruded Mg-Li-Al-Zn-Si alloy can be expressed as follows:

(7) $$\dot{\varepsilon }\exp\left(\frac{138,643}{RT}\right)=e^{29.6}{\left[\sinh\left(0.0211\sigma \right)\right]}^{4.143},$$

**Figure 7:**
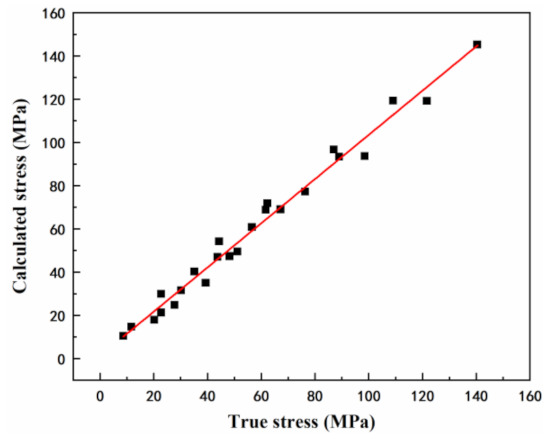
Correlation between experimental and predicted peak stress data from constitutive equation.

Figure 7 shows the comparison of the peak stress between the experimental and predicted results by the developed constitutive equations of the as-extruded Mg-Li-Al-Zn-Si alloy at different temperatures under strain rates of 0.01, 0.1, 1, 10 s^−1^. The correlation coefficient was used to quantify the predictability of the peak stress and was calculated as 0.992.

**Figure 8:**
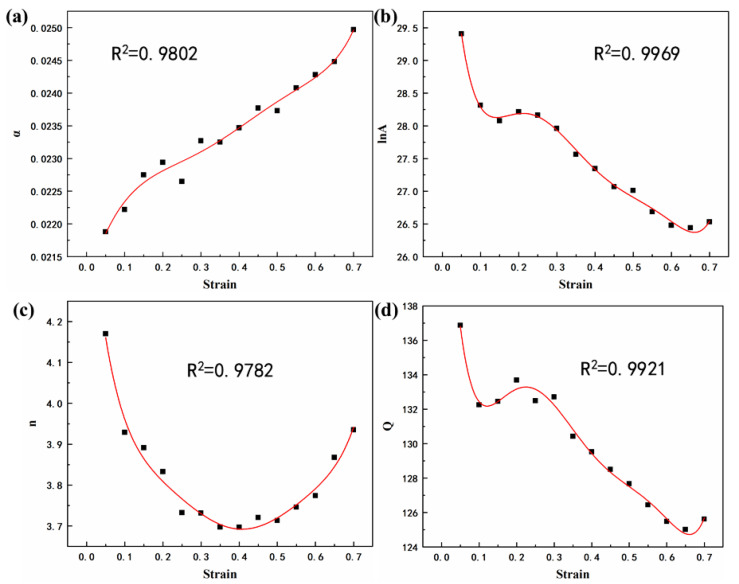
Relationships between: (**a**) *α*, (**b**) ln*A*, (**c**) *n*, (**d**) *Q* and true strain by polynomial fit of Mg-Li-Al-Zn-Si alloy.

As Figure 4 shows, the effect of strain is significant in regimes with a lower working temperature. To improve the applicability of the constitutive equation, compensation of strain should be taken into account [25,26]. Assuming that the material constants are a polynomial function of the strains, as shown in Equation (8), then the values of material constants (*α*, *A*, *n*, *Q*) of the constitutive equation were computed under different strains in the range of 0.05-0.7, with the interval of 0.05. The polynomial fit results of *α*, ln*A*, n, *Q* of the Mg-Li-Al-Zn-Si alloy are provided in Table 2 and the relationships between *α*, ln*A*, *n*, *Q*, and the true strain are shown in Figure 8. The fitted curves are strongly correlative to the values of the constants.

(8) $$\left\{\begin{array}{l} \alpha =\alpha_{0}+\alpha_{1}\varepsilon +\alpha_{2}\varepsilon^{2}+\alpha_{3}\varepsilon^{3}+\alpha_{4}\varepsilon^{4}+\alpha_{5}\varepsilon^{5}+\alpha_{6}\varepsilon^{6}\\ \ln A=A_{0}+A_{1}\varepsilon +A_{2}\varepsilon^{2}+A_{3}\varepsilon^{3}+A_{4}\varepsilon^{4}+A_{5}\varepsilon^{5}+A_{6}\varepsilon^{6}\\ n=n_{0}+n_{1}\varepsilon +n_{2}\varepsilon^{2}+n_{3}\varepsilon^{3}+n_{4}\varepsilon^{4}+n_{5}\varepsilon^{5}+n_{6}\varepsilon^{6}\\ Q=Q_{0}+Q_{1}\varepsilon +Q_{2}\varepsilon^{2}+Q_{3}\varepsilon^{3}+Q_{4}\varepsilon^{4}+Q_{5}\varepsilon^{5}+Q_{6}\varepsilon^{6} \end{array}\right.$$

**Figure 9:**
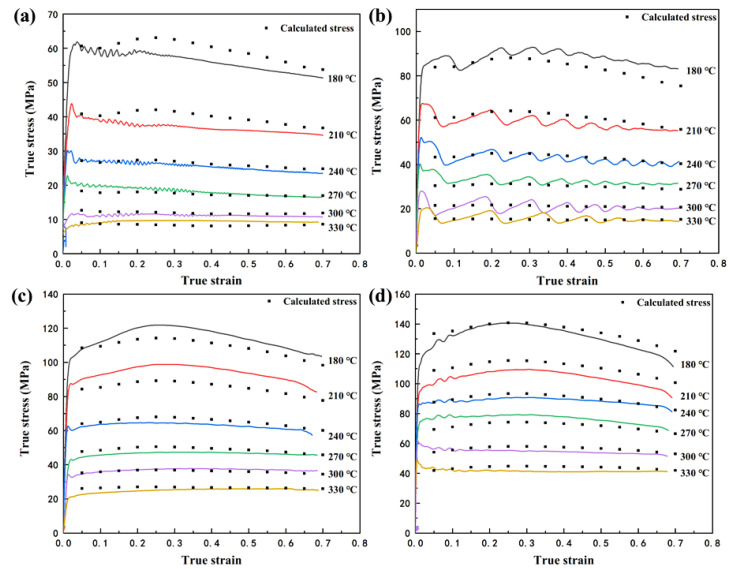
Comparisons between predicted and measured flow stress curves of Mg-Li-Al-Zn-Si alloy deformed to a strain of 0.7 at strain rates of (**a**) 0.01 s^−1^, (**b**) 0.1 s^−1^, (**c**) 1 s^−1^ and (**d**) 10 s^−1^ with different temperatures.

To confirm the accuracy of the constitutive equation with relation to the compensation of strain, a comparison between the calculated and the experimental stresses was performed. The flow stress values, which deformed at 180–330 °C/0.01–10 s^−1^ under different strains, in the range of 0.05–0.7 with the interval of 0.05 were calculated from the above strain-compensation constitutive equation. Figure 9 shows the distribution of the calculated datapoints. One can determine that the calculated and experimental values are in good agreement above 240 °C, and there are significant errors between the datapoints and curves below 240 °C.

### 3.4. Processing Map

#### 3.4.1. Establishment of Processing Map

The workability of a material is determined by chemical composition, microstructural characteristic, deformation path, and thermal processing parameters. Therefore, the processing map can be used to analyze the thermoformabilities and the deformation mechanism, under different deformation conditions, and to optimize the thermal processing parameters to avoid the occurrence of defects. The processing maps are established based on the dynamic materials model (DMM), which was presented by Prasad and Gegel [27]. The dynamic materials model regards the thermal deformed material as a nonlinear power dissipator and the material processing of the material as an energy dissipation system. The input power (represented by *P*) is consumed in two ways: the plastic deformation heat (represented by *G*) and the microstructural evolution (represented by *J*). This can be defined as [28]:

(9) $$P=\sigma \dot{\varepsilon }=G+J=\int_{0}^{\dot{\varepsilon }}\sigma d\dot{\varepsilon }+\int_{0}^{\sigma }\dot{\varepsilon }d\sigma ,$$

the ratio of *G* and *J* is determined by the strain rate sensitivity (m), m is material constant and can be described as [29]:

(10) $$m=\frac{\partial J}{\partial G}=\frac{\dot{\varepsilon }\partial \sigma }{\sigma \partial \dot{\varepsilon }}=\frac{\partial \left(\ln\sigma \right)}{\partial \left(\ln\dot{\varepsilon }\right)},$$

assuming the flow behavior of the material follows a power-law equation:

(11) $$\sigma =k\cdot {\dot{\varepsilon }}^{m},$$

then *J* can be expressed by:

(12) $$J=\int_{0}^{\dot{\varepsilon }}\sigma d\dot{\varepsilon }=\frac{m}{m+1}\sigma \dot{\varepsilon }$$

The proportion between the energy dissipated in microstructure evolution and the linear energy dissipated (*m* = 1) is defined as efficiency of power dissipation (*η*):

(13) $$\eta =\frac{J}{J_{\max}}=\frac{2m}{2m+1}$$

**Figure 10:**
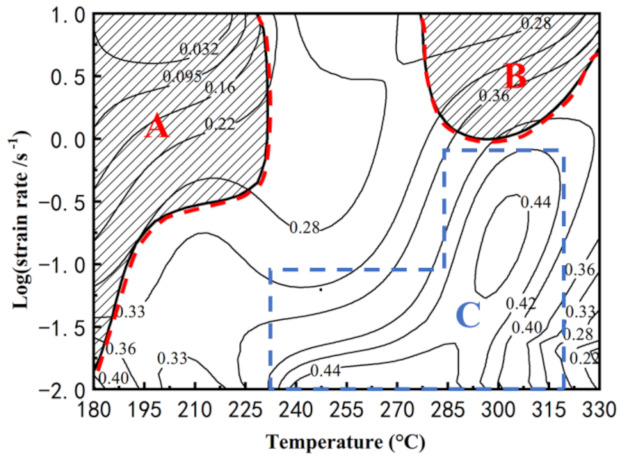
The processing map of Mg-Li-Al-Zn-Si alloy developed at strain of 0.7.

According to the extremum principle of irreversible thermodynamics, several instability criteria were established. Based on the maximum entropy production principle, the criterion presented by Prasad is expressed as follows [30]:

(14) $$\xi \left(\dot{\varepsilon }\right)=\frac{\partial \ln\left[m/\left(m+1\right)\right]}{\partial \ln\dot{\varepsilon }}+m<0,$$

where *ξ* is the instability parameter. Figure 10 shows the processing map of the Mg-Li-Al-Zn-Si alloy at the strain of 0.7 in the temperature range of 180–330 °C and strain rate range of 0.01–10 s^−1^, which is developed by the supposition of the power dissipation map and the instability map. The shadows represent the flow instability domain and the contour numbers represent the efficiency of power dissipation. The peak power dissipation efficiency is 0.44 under the processing parameters of 0.1 s^−1^/300 °C and 0.01 s^−1^/270 °C. The instability domains lie in the temperature range of 180–230 °C and 280–330 °C with high strain rates.

#### 3.4.2. Microstructural Analysis Based on the Processing Map

The domains marked in Figure 10 can be interpreted based on the power dissipation efficiency and the relationship with microstructural evolution. Domain A and B are unstable regions that lie within the low-temperature range (180–230 °C) and the high-temperature range (280–330 °C), respectively. Conversely, domain A covers a larger range of strain rates. Domain A and B are unsuitable as deformation regions that prevent deformation defects. Domain C spreads over the temperature and strain rate range of 240–320 °C and 0.01–1 s^−1^ with high power dissipation efficiency. For the low stacking fault energy alloys, there is evidence that domains with a power dissipation efficiency of 30–50% are liable for DRX processes [31]. The dominant mechanism in each region is investigated by microstructural evolution.

**Figure 11:**
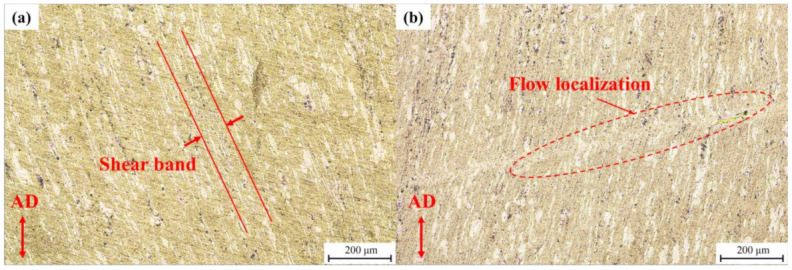
Optical micrographs for the deformed samples at different deformation conditions at instability region: (**a**) 180 °C, 0.01 s^−1^, (**b**) 180 °C, 0.1 s^−1^.

**Figure 12:**
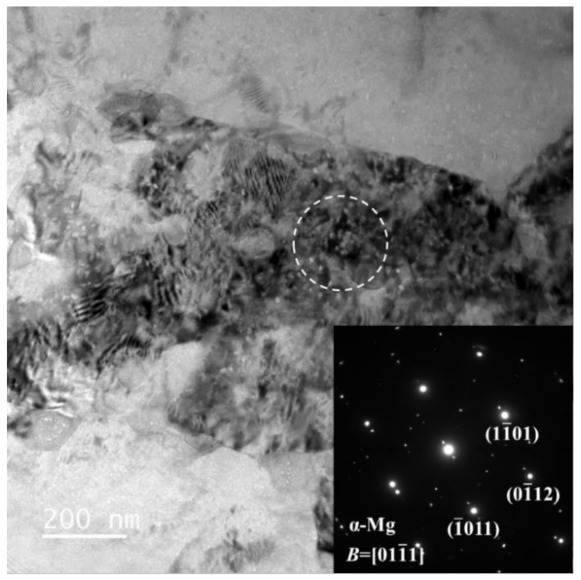
TEM images of specimen deformed at 180 °C with the strain rate of 10 s^−1^.

Domain A is an instability region at a low temperature. The typical microstructure of the alloy deformed at 180 °C, with a strain rate of 0.01 s^−1^ and 0.1 s^−1^, is shown in Figure 11. Strong strip structure, which has a certain angle to the axis of compression leading to the formation of shear bands, is shown in Figure 11a. As Figure 11b shows, the microstructure deformed at 180 °C and 0.1 s^−1^ exhibits a flow localization, which may cause microcracking and a feasible deformation zone [32]. In many cases, due to the differences between the ductility of the two phases, the strain/stress-bearing of the phases reflects a synergistic interaction of their properties [33,34]. The contribution of strain hardening and high local stress concentrations, caused by the plastic incompatibility between the phases, lead to the local instability. The TEM image of the specimen deformed at 180 °C with a strain rate of 10 s^−1^ is shown in Figure 12. Compared with the β-phase, the dislocation density in the α-phase is high. Due to the HCP structure, the dislocation glide and climb is particularly difficult, resulting in a dislocation pile-up in α-phase grains.

**Figure 13:**
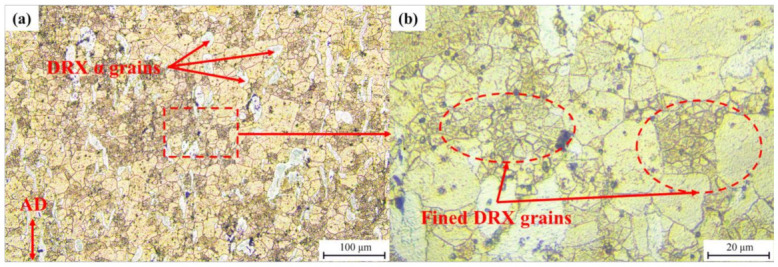
Representative microstructure characteristics of specimen deformed at 330 °C with the strain rate of 10 s^−1^. (**a**) DRX of dual-phase; (**b**) uneven microstructure.

Domain B lies in the temperature range of 280–330 °C and the strain rate range of 1–10 s^−1^. With a power dissipation efficiency of around 0.3, the microstructure deformed at the condition of 330 °C/10 s^−1^ is shown in Figure 13. As Figure 13a shows, a considerable amount of refined α-phase DRX grains are surrounded by growth β-phase DRX grains. Part of α-phases transform into the approximately globular grains. The grain boundaries of the untransformed α-phase grains remain as a uniform vector in the radial direction owing to the weak recovery of grain boundary torsion [35]. Meanwhile, the glomeration morphology of the α-phase occurred during the deformation, which is considered responsible for the flow softening [36]. The mechanism of softening of Mg-Li duplex phase alloys is not only attributed to DRX but also to the glomeration of α-phase [37]. An inhomogeneous microstructure of β-phase is shown in Figure 13, which is composed of large grains (17.85 μm) and a few relatively fine grains (3.05 μm). Due to the high deformation temperature and the strain rate, the process of DRX in β-phase seems to be complete. It can be concluded that the formation of some excessively coarse grains, surrounded by DRXed finer grains, is the result of an abnormal grain growth. Similar phenomena have been observed in the investigation of an extruded LZ121 alloy [38,39]. This type of microstructure is undesirable in the hot working process and should be avoided.

**Figure 14:**
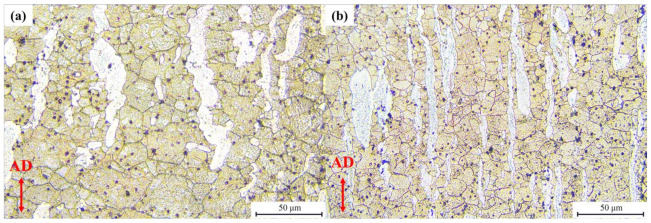
Images of typical microstructure of Mg-Li-Al-Zn-Si alloy deformed with the condition of (**a**) 270 °C, 0.01 s^−1^, (**b**) 300 °C, 0.1 s^−1^.

Domain C is the high value of the power dissipation efficiency region. With the peak power dissipation efficiency of 0.44, the microstructure obtained at the deformation condition of 270 °C/0.01 s^−1^ and 300 °C/0.1 s^−1^ is shown in Figure 14a,b, respectively. With the exception of newly formed DRX grains from the α-phase, the grain boundaries of the microstructure deformed at 270 °C/0.01 s^−1^ are formed via dislocation rearrangement. Therefore, the grain boundaries of the α-phase are deformed and curved. Typically, CDRX occurs in the deformed region, with a high dislocation density and is favored at low temperatures. The increase in temperature will enhance dislocation recovery, slowing down the CDRX [40]. However, in related studies, strain is an important factor that affects CDRX, acting as an enabler [41,42]. As the deformation condition changes to 300 °C/0.1 s^−1^, most α-phases maintain the elongated microstructure without shear bands. Due to the relatively low strength of the α-phase, plastic deformation occurs first in the β-phase. The stored energy in the β-phase will increase. Therefore, DRX preferentially occurs in the β-phase. The same phenomenon has been detected in the Mg-8Li-3Al-2Zn-0.2Zr alloy [43]. One can observe that the β-phase developed many equiaxed grains, together with some finer grains. The average grain sizes of the β-phase in Figure 14a,b are about 15.37 μm and 9.16 μm, respectively. The formation of a considerable amount of equiaxed grains proves that DRX has comprehensively occurred [44].

**Figure 15:**
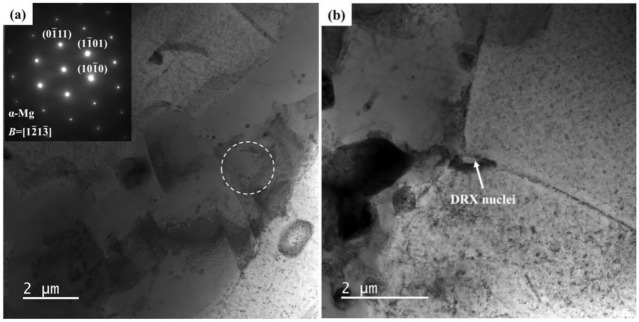
TEM images of specimen deformed at 270 °C with the strain rate of 0.01 s^−1^ (**a**) subgrain boundaries, (**b**) DRX nuclei formed at triple grain boundary.

To further investigate the microstructure evolution of the DRX process, the TEM micrographs of specimens deformed at 270 °C/0.01 s^−1^ are shown in Figure 15. Figure 15a shows that the grain boundaries of the α-phase grains are irregular. The dislocation density in the grain interior is lower than that of the specimen deformed at 180 °C/10 s^−1^. The original grain is subdivided by some developing subgrain boundaries. Through deformation-induced dislocation accumulation and recombination, the dislocation cells start to generate in the original grains. During the deformation process, the dislocation cell boundaries are transformed into low-angle subgrain boundaries. The appearance of transformation from the low-angle boundaries to the high-angle boundaries occurs with the increased strain [45]. Such a morphology of the subgrain boundaries confirms that the mechanism of the DRX belongs to the CDRX process [46]. As Figure 15b illustrates, the grain boundaries of the β-phase are sharp and there are few dislocations inside the grains, which are typical attributes of grains generated by DDRX [47]. The triple point serves as a prominent nucleation site for a DRX grain and there are few dislocations in grains. At the early stage, the entire DRX nucleus represents a grain boundary region. During the process of deformation, the DRX grain boundary region progressively travels outwards [48]. With the strain rate of 0.1 s^−1^, the time for the DRX process is relatively short. As a result, the DRX nuclei could not entirely grow.

## 4. Conclusions

The hot deformation behavior of the Mg-Li-Al-Zn-Si alloy was studied through constitutive equations and processing maps through a hot compression test and further observation of microstructural characteristics. The main conclusions, based on the experiments and analyses, are summarized below.

(1) At the temperature range of 180–330 °C and strain rate range of 0.01–10 s^−1^, the flow-stress–true-strain curves for the Mg-Li-Al-Zn-Si alloy are sensitive to the deformation temperature and strain rate. The shape transformation from the multiple to single peak of the flow-stress–true-strain curves with the increasing strain rate was caused by the DRX cycle.
(2) The constitutive equation was found to precisely predict flow stress at high temperatures (>240 °C) but showed significant deviation at low temperatures.
(3) The processing map based on DMM at the strain of 0.7 was established for the Mg-Li-Al-Zn-Si alloy. The peak power dissipation efficiency is 0.44 when the deformation conditions are 300 °C/0.1 s^−1^ and 270 °C/0.01 s^−1^. The unsafe domains are detected at low temperatures (<230 °C) and high temperatures (>280 °C) with high strain rates (>1 s^−1^) that should be avoided.
(4) The dominant nucleation mechanism of DRX in the safe region of the Mg-Li-Al-Zn-Si alloy is different in two phases. In the α-phase, CDRX occurs with the accumulation of dislocations in subgrain boundaries, leading to the increase in their orientation. The microstructure of the β-phase exhibits a DDRX character.

## Figures and Tables

**Table 1:** The element contents of the Mg-Li-Al-Zn-Si alloy (wt.%).

Li	Al	Zn	Si	Mg
10.10	2.98	3.12	0.22	Bal.

**Table 2:** Polynomial fit results of *α*, *n*, *Q*, and ln*A* of Mg-Li-Al-Zn-Si alloy.

*α*	Value	*N*	Value	*Q*	Value/(kJ/mol)	ln*A*	Value
*α*_0_	0.021	*n*_0_	4.580	*Q*_0_	152.508	*A*_0_	32.906
*α*_1_	0.020	*n*_1_	−11.689	*Q*_1_	−495.219	*A*_1_	−107.133
*α*_2_	−0.088	*n*_2_	80.028	*Q*_2_	4475.528	*A*_2_	914.041
*α*_3_	0.173	*n*_3_	−308.404	*Q*_3_	−18,940.418	*A*_3_	−3753.913
*α*_4_	−0.019	*n*_4_	644.671	*Q*_4_	40,466.631	*A*_4_	7862.544
*α*_5_	−0.311	*n*_5_	−677.602	*Q*_5_	−42,731.404	*A*_5_	−8179.457
*α*_6_	0.258	*n*_6_	282.294	*Q*_6_	17,758.101	*A*_6_	3359.740

## Data Availability

All data are available from the corresponding author on reasonable request.

## References

[1] Haferkamp H., Niemeyer M., Boehm R. (2000). Development, processing and applications range of magnesium lithium alloys. Mater. Sci. Forum..

[2] Park G.H., Kim J.T., Park H.J., Kim Y.S., Jeong H.J., Lee N., Seo Y., Suh J.Y., Son H.T., Wang W.M. (2016). Development of lightweight Mg-Li-Al alloys with high specific strength. J. Alloys Compd..

[3] Wang J., Zhang M., Shi B., Zhang L., Jin P. (2020). Ex-situ EBSD investigation of the reduced c/a values and work hardening behavior in Mg-4Li-1Al-0.5Y alloy under hot compression. Mater. Sci. Eng. A.

[4] Drozd Z., Trojanova Z., Kúdela S. (2004). Deformation behavior of Mg-Li-Al alloys. J. Alloys Compd..

[5] Zhao J., Zhang J., Liu W., Liang Z. (2016). Effect of Y content on microstructure and mechanical properties of as-cast Mg-8Li-3Al-2Zn alloy with duplex structure. Mater. Sci. Eng. A.

[6] Nr A., Sg B., Sk C. (2018). Mechanical behavior of Mg-Li-Al alloys. Mater. Today Proc..

[7] Li C.Q., Liu X., Dong L.J., Shi B.Q., Zhang Z.R. (2021). Simultaneously improved mechanical strength and corrosion resistance of Mg-Li-Al alloy by solid solution treatment. Mater. Lett..

[8] Dobkowska A., Adamczyk–Cieślak B., Kuc D., Hadasik E., Plocinski T., Ura-Bińczyk E., Mizera J. (2021). Influence of bimodal grain size distribution on the corrosion resistance of Mg-4Li-3Al-1Zn (LAZ431). J. Mater. Res. Technol..

[9] Shao B., Wu S., Shan D., Guo B., Zong Y. (2019). The effect of thermal cycling process between high and low temperatures on the microstructure and properties of Mg-10Li-3Al-3Zn-0.25Si alloy. Mater. Lett..

[10] Askariani S.A., Pishbin S.H. (2016). Hot deformation behavior of Mg-4Li-1Al alloy via hot compression tests. J. Alloys Compd..

[11] Li X., Ren L., Le Q., Jin P., Li D. (2020). The hot deformation behavior, microstructure evolution and texture types of as-cast Mg-Li alloy. J. Alloys Compd..

[12] Ion S.E., Humphreys F.J., White S.H. (1982). Dynamic recrystallisation and the development of microstructure during the high temperature deformation of magnesium. Acta Metall..

[13] Yang Y., Peng X.D., Wen H.M., Zheng B., Zhou Y. (2013). Influence of extrusion on the microstructure and mechanical behavior of Mg-9Li-3Al-xSr alloys. Metall. Mater. Trans. A.

[14] Liang X.L., Peng X., Hao J.I., Liu W.C., Wu G.H., Ding W.J. (2021). Microstructure and mechanical properties of as-cast and solid solution treated Mg-8Li-xAl-yZn alloys. Trans. Nonferrous Met. Soc. China.

[15] Zhang C., Liang W.U., Zhao Z.L., Xie Z.H., Pan F.S. (2019). Effect of li content on microstructure and mechanical property of mgxli3(alsi) alloys. Trans. Nonferrous Met. Soc. China..

[16] Ling W. (2007). Effect of neodymium on microstructure and corrosion resistance of AZ91 magnesium alloy. J. Mater. Sci..

[17] Liang C., Zhao G., Yu J. (2015). Hot deformation behavior and constitutive modeling homogenized 6026 aluminum alloy. Mater. Des..

[18] Kwak T.Y., Lim H.K., Kim W.J. (2015). Hot compression characteristics and processing maps of a cast Mg-9.5Zn-2.0Y alloy with icosahedral quasicrystalline phase. J. Alloys Compd..

[19] Takaki T., Hisakuni Y., Hirouchi T., Yamanaka A., Tomita Y. (2009). Multi-phase-field simulations for dynamic recrystallization. Comput. Mater. Sci..

[20] Kugler G., Turk R. (2004). Modeling the dynamic recrystallization under multi-stage hot deformation. Acta Mater..

[21] Prasad Y., Rao K.P. (2005). Processing maps and rate controlling mechanisms of hot deformation of electrolytic tough pitch copper in the temperature range 300–950 °C. Mater. Sci. Eng. A.

[22] Sakai T., Jonas J.J. (1984). Overview no. 35 dynamic recrystallization: Mechanical and microstructural considerations. Acta Metall..

[23] Jonas J.J., Sellars C.M., Tegart W.J. (1969). Strength and structure under hot-working conditions. Metall. Rev..

[24] Zener C., Hollomon J.H. (1944). Effect of strain rate upon plastic flow of steel. J. Phys. D.

[25] Lin Y.C., Chen M.S., Zhong J. (2008). Constitutive modeling for elevated temperature flow behavior of 42CrMo steel. Comput. Mater. Sci..

[26] Jiang L., Li F., Cai J., Wang R., Yuan Z., Xue F. (2012). Flow behavior modeling of the 7050 aluminum alloy at elevated temperatures considering the compensation of strain. Mater. Des..

[27] Prasad Y.V., Gegel H.L., Doraivelu S.M., Malas J.C., Morgan J.T., Lark K.A., Barker D.R. (1984). Modeling of dynamic material behavior in hot deformation: Forging of Ti-6242. Metall. Trans. A.

[28] Ziegler H. (1966). Some extremum principles in irreversible thermodynamics with applications to continuum mechanics. Prog. Solid Mech..

[29] Prasad Y.V.R.K. (2003). Processing maps: A status report. J. Mater. Eng. Perform..

[30] Prasad Y. (1990). Recent advances in the science of mechanical processing. Indian J. Technol..

[31] Wang J., Dong J., Zhang M., Xie X. (2013). Hot working characteristics of nickel-base superalloy 740h during compression. Mater. Sci. Eng. A.

[32] Jenab A., Taheri A.K. (2014). Experimental investigation of the hot deformation behavior of AA7075: Development and comparison of flow localization parameter and dynamic material model processing maps. Int. J. Mech. Sci..

[33] Yi H.L., Shu M.W., Fang H.K., Shing H.W., Jer R.Y., Chia C.Y., Chuan S.C. (2011). Microtwin formation in the α phase of duplex titanium alloys affected by strain rate. Mater. Sci. Eng. A.

[34] Farabi E., Zarei-Hanzaki A., Pishbin M.H., Moallemi M. (2015). Rationalization of duplex brass hot deformation behavior: The role of microstructural components. Mater. Sci. Eng. A.

[35] Xia Y.F., Liu Q., Gao L., Zhou J., Wu D.S., Luo G.C., Quan G.Z. (2014). Dynamic recrystallization kinetics in α phase of as-cast Ti-6Al-2Zr-1Mo-1V alloy during compression at different temperatures and strain rates. Mater. Sci. Eng. A.

[36] Momeni A., Abbasi S.M. (2010). Effect of hot working on flow behavior of Ti-6Al-4V alloy in single phase and two phase regions. Mater. Des..

[37] Xu T.C., Peng X.D., Qin J., Chen Y.F., Yang Y., Wei G.B. (2015). Dynamic recrystallization behavior of Mg-Li-Al-Nd duplex alloy during hot compression. J. Alloys Compd..

[38] Karami M., Mahmudi R. (2013). The microstructural, textural, and mechanical properties of extruded and equal channel angularly pressed Mg-Li-Zn alloys. Metall. Mater. Trans. A.

[39] Karami M., Mahmudi R. (2014). Work hardening behavior of the extruded and equal-channel angularly pressed Mg-Li-Zn alloys under tensile and shear deformation modes. Mater. Sci. Eng. A.

[40] Wen H.M., Zhao Y.H., Topping T.D. (2012). Influence of pressing temperature on microstructure evolution and mechanical behavior of ultrafine-grained Cu processed by equal-channel angular pressing. Adv. Eng. Mater..

[41] Yang X., Okabe Y., Miura H., Sakai T. (2012). Effect of prior strain on continuous recrystallization in AZ31 magnesium alloy after hot deformation. Mater. Sci. Eng. A.

[42] Miura H., Maruoka T., Jonas J.J. (2013). Effect of ageing on microstructure and mechanical properties of a multi-directionally forged Mg-6Al-1Zn alloy. Mater. Sci. Eng. A.

[43] Sun Y., Wang R., Ren J., Peng C., Feng Y. (2019). Hot deformation behavior of Mg-8Li-3Al-2Zn-0.2Zr alloy based on constitutive analysis, dynamic recrystallization kinetics, and processing map. Mech. Mater..

[44] Karami M., Mahmudi R. (2014). Hot shear deformation constitutive analysis and processing map of extruded Mg-12Li-1Zn bcc alloy. Mater. Des..

[45] Gourdet S., Montheillet F. (2000). An experimental study of the recrystallization mechanism during hot deformation of aluminium. Mater. Sci. Eng. A.

[46] Sakai T., Miura H., Goloborodko A., Stitdikov O. (2009). Continuous dynamic recrystallization during the transient severe deformation of aluminum alloy 7475. Acta Mater..

[47] Mcqueen H.J. (2004). Development of dynamic recrystallization theory. Mater. Sci. Eng. A.

[48] Beladi H., Cizek P., Hodgson P.D. (2010). On the characteristics of substructure development through dynamic recrystallization. Acta Mater..

